# Overexpression of miR-199b-5p inhibits Ewing's sarcoma cell lines by targeting CCNL1

**DOI:** 10.3892/mmr.2015.3888

**Published:** 2015-06-03

**Authors:** WEIHUA LI, YUXIA LI, JIANKUO GUO, HUAGANG PAN, YONGLE ZHANG, XIAO WANG

**Affiliations:** 1 Department of Orthopedic Surgery, Henan University Hospital of Huaihe Henan University Clinical College, Kaifeng, Henan 475000, P.R. China; 2 Clinical Laboratory, Henan University Hospital of Huaihe Henan University Clinical College, Kaifeng, Henan 475000, P.R. China

**Keywords:** Ewing's sarcoma, miR-199b-5p, CCNL1

## Abstract

MicroRNAs (miRNAs) are known to regulate the expression of a variety of genes, which are important in the development of several types of tumor, including Ewing's sarcoma (ES), at the post-transcriptional level. Although previous studies have identified that the expression of miRNA-199b-5p was downregulated in various types of tumor, the expression levels of miR-199b-5p in ES cells remain to be elucidated. The mechanism underlying ES via the miRNA pathway remains to be elucidated. The present study demonstrated that miR-199b-5p was an important regulator in ES cells and its expression was downregulated in ES originated A673/TC252 cells. The ES cell lines, A673 and TC252, were transfected with an miR-199b-5p mimic to overexpress the levels of this miRNA. This forced expression of miR-199b-5p suppressed the cell proliferation and invasion, arrested cell cycle progression, and promoted cell apoptosis. Furthermore, CCNL1 was identified by bioinformatic software as a potential target gene of miR-199b-5p. Following this, the present study identified CCNL1 as a direct target of miR-199b-5p in ES cells. Taken together, the present study established a functional link between ES, miR-199b-5p and CCNL1, and suggested that miR-199b-5p acts as a tumor suppressor and may be of diagnostic and therapeutic importance for human ES.

## Introduction

Ewing's sarcoma (ES) is a highly malignant bone tumor occurring in children, adolescents and young adults (1), which is a type of primitive neural ectoderm family of invasive tumor (2). Patients with ES under the age of 15 comprise ~80% of ES cases (3). Currently, clinical chemotherapy, surgery and radiotherapy, including traditional methods, are used to treat ES. However, only 60% of the patients with local disease are cured (4,5). The underlying mechanism of ES remains to be elucidated.

MicroRNAs (miRNAs) are a type of endogenous non-coding small RNA molecule, ~22 nucleotides in length, which regulate the expression of target genes predominantly at the post-transcriptional level (6,7). Previous studies demonstrated that miRNAs were involved in almost all the human physiological activities, including cell proliferation, apoptosis, cell growth and differentiation, and provided increasing confirmation in these aspects, and at the same time also suggested that miRNAs are involved in the occurrence of various types of disease (8,9). Revealing the regulatory function and mechanism of miRNAs may assist in better understanding the complex regulatory network of higher eukaryotes. This may lead to miRNAs being used clinically as convenient and practical gene therapy treatments.

miRNAs were demonstrated to be important in ES cells (10,11). The present study used ES cell lines, A673 and TC252, to investigate the function and mechanism of miR-199b-5p *in vitro*. It was demonstrated that miR-199b-5p was a tumor suppressor in these ES cell lines, which inhibited cell proliferation and cell invasion, arrested cell cycle progression, and promoted apoptosis. In addition, it was revealed that miR-199b-5p directly targeted CCNL1 to perform this function in ES cells. Taken together, the present study successfully identified miR-199b-5p as a key regulator in ES cells.

## Materials and methods

### Cell lines and cell culture

The A673 and TC252 cell lines were maintained in RPMI-1640 (HyClone Corp., Logan, UT, USA), supplemented with 10% fetal bovine serum (FBS; PAA, Cölbe, Germany). The human mesenchymal stem cells (MSCs) as well as the A673 and TC252 cell lines were obtained from the American Type Culture Collection (ATCC; Rockville, MD, USA) and were cultured in Iscove's modified Eagle's medium, supplemented with 10% FBS and platelet-derived growth factor-BB (10 ng/ml). The 293T cells were obtained from ATCC and cultured in Dulbecco's modified Eagle's medium, supplemented with 10% FBS.

### Oligonucleotides and transfection

The miRNA-199b-5p mimic and scramble control molecules were obtained from Dharmacon (Chicago, IL, USA) and were transfected into the A673 and TC252 cells at a final concentration of 60 nM. The A673 and TC252 cells were mixed into complete medium following 6 h culture and were washed with phosphate-buffered saline (PBS) 48 h following transfection for the subsequent experiments.

### RNA extraction and reverse transcription quantitative polymerase chain reaction (RT-qPCR)

The total RNA was extracted using TRIzol reagent (Invitrogen Life Technologies, Carlsbad, CA, USA), according to the manufacturer's instructions. To determine the quality and purity of the RNA, the absorption peak of RNA was detected at 260 nm, 260/280 nm, and 260/230 nm using a NanoDrop (NanoDrop Technologies,. Inc., Thermo Fisher Scientific, Wilmington, DE, USA). The cDNA was generated using M-MLV reverse transcriptase (Invitrogen Life Technologies), according to the manufacturer's instructions.

RT-qPCR was performed using the SYBR Premix ExTaq kit (Takara Bio, Inc., Dalian, China) on the ABI PRISM 7500 real-time PCR System (Applied Biosystems, Foster City, CA, USA). All oligonucleotides were transfected into A673 and TC252 cells using Dhamafect 1 (Dharmacon). The sequences of the specific primers used for PCR were: miR-199b-5P, forward: 5′-CAG CCC AGT GTT TAG ACT ATC-3′ and reverse: 5′-CAG TGC AGG GTC CGA GGT-3′; U6, forward: 5′-CTC GCT TCG GCA GCA CAT ATACT-3′ and reverse: 5′-ACG CTT CAC GAA TTT GCG TGTC-3′. The cycling conditions were as follows: 95°C for 3 min, 95°C for 5 sec, 60°C for 30 sec, 72°C for 30 sec then 72°C for 10 min for 30 cycles. The data were uniformly normalized to the internal control U6 and the relative expression levels were evaluated using the 2-ΔΔCt method (12). The mRNA expression levels of U6 were used as endogenous control. All the experiments were performed three times.

### Proliferation assay

The cells were seeded (8×10^3^ cells/well) in 24-well plates and proliferation was determined using a cell counting kit-8 (CCK8; Dojindo Laboratories, Kumamoto, Japan). miR-99b-5p mimic was transfected after 16 h. The cells were collected and digested with 0.25% trypsin, then, suspended in fresh medium. The proliferative activity was detected at 0, 24, 48 and 72 h with CCK8. A total of 10 *µ*l/well CCK8 was added to the medium 2 h prior to testing at 37°C. The absorbance in each well was measured with a microplate reader at 450 and 630 nM. (650 nm wavelength as reference wavelength; Bio-Rad Laboratories, Inc., Hercules, CA, USA).

### Cell cycle analysis

The A673 and TC252 cells were cultured in serum-free medium following 4 h of transfection and were cultured for 24 h, prior to the addition of complete medium. The A673 and TC252 cells were washed twice with cold PBS 48 h following transfection and were subsequently fixed in cold 70% alcohol overnight. The fixed cells were incubated in propidium iodide and RNase A, and were detected by flow cytometry using a C6 Flow Cytometer^®^ Instrument (BD Biosciences, Franklin Lakes, NJ, USA).

### Apoptosis assay

The A673 and TC252 cells were labeled with Annexin-V at early apoptosis and were labeled with 7AAD at late apoptosis or to indicate cell death. The collected cells were diluted to 5×10^5^ cells/ml 48 h after transfection. The cells were washed twice with cold PBS and were subsequently incubated with PE annexin-V and 7AAD (BD Biosciences, Bedford, MA, USA). The data were analysis by fluorescence-activated cell sorting.

### Invasion assays

The A673 and TC252 cells were collected and adjusted at a concentration of 2×10^5^ cells/ml 24 h after transfection. Culture medium, containing 10% FBS, was added to the lower chamber (at the bottom of a 24-well plate) and the cell suspension was added to the upper chamber. The A673 and TC252 cells were maintained in culture for 24 h. The cells in the upper chamber were washed away and stained with 0.1% crystal violet at room temperature for 15 min. The number of cells were counted in 10 different fields under the microscope (CKX41; Olympus, Tokyo, Japan) and the total number of cells invading through the Matrigel were counted in 10 representative fields under a microscope (BD Biosciences).

### Western blot analysis

The A673 and TC252 cells were collected 48 h after transfection, washed twice with PBS and lysed on ice in cold modified radioimmunoprecipitation buffer supplemented with protease inhibitors (Roche, Mannheim, Germany) for 30 min. The protein concentration was detected using a Bicinchoninic Acid Protein Assay kit (Beyotime Institute of Biotechnology, Shanghai, China). An equal quantity of protein (30 *µ*g) was separated by 10% SDS-PAGE. The protein was transferred onto a nitrocellulose membrane (Millipore, Billerica, MA, USA) following electrophoresis. The membrane was blocked for 2 h with 5% non-fat milk and was incubated with antibodies against CCNL1 (1:200; cat. no. abc-102; Millipore), c-kit (1:100; cat. no. ab5506; Abcam, Cambridge, MA, USA) and GAPDH (1:5,000; Cell Signaling Technology, Inc., Danvers, MA, USA) overnight at 4°C. Following incuabtion with a secondary antibody (cat. no. ZB-2301; Zhong-Shan JinQuao, Shanghai, China), detection was performed using an enhanced chemiluminescence system (Thermo Fisher Scientific, Waltham, MA, USA).

### Statistical analysis

The data were analyzed by the Student's t-test (two-tailed). P<0.05 was considered to indicate a statistically significant difference.

## Results

### Expression of miR-199b-5p is downregulated in ES cells

**Figure 1:**
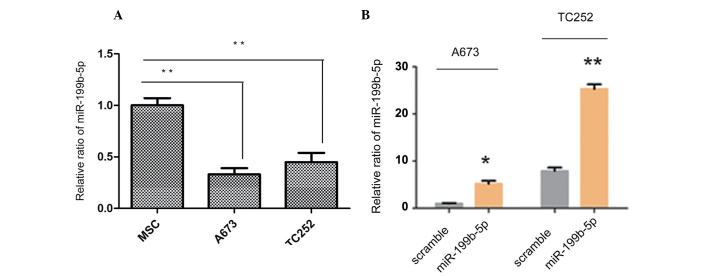
Expression of miR-199b-5p in ES cell lines. (A) The expression levels of miR-199b-5p in ES cell lines were significantly decreased compared with mesenchymal stem cells, as detected by reverse transcription quantitative polymerase chain reaction. (B) Forced expression of miR-199b-5p significantly upregulated the expression levels of miR-199b-5p compared with the scramble control. (^*^P<0.05, ^**^P<0.01, compared with scramble cells). ES, Ewing's sarcoma; miR, microRNA.

RT-qPCR revealed that the expression levels of miR-199b-5p were downregulated in the ES cell line compared with the human MSCs, as shown in Fig. 1A. The MSC bone marrow mesenchymal stem cells were used as normal scramble control ES cells. A mature miR-199b-5p mimic and scramble mimic were constructed and transfected into the cells *in vitro* to overexpress the levels of miR-199b-5p in the A673 and TC252 cells. The expression levels of the miR-199b-5p mimic were subsequently detected in the A673 and TC252 cells. As shown in Fig. 1B, the overexpression was considered significantly different, compared with the scramble control. Taken together, these findings suggested that miR-199b-5p may act as a negative modulator in ES cells.

### Overexpression of miR-199b-5p inhibits proliferation and invasion, inhibits cell cycle progression, and induces apoptosis in ES cells

**Figure 2:**
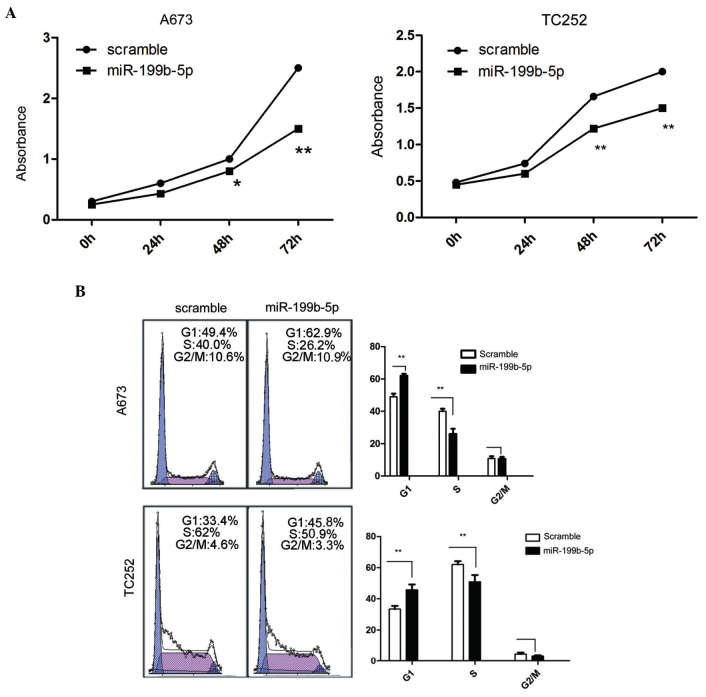

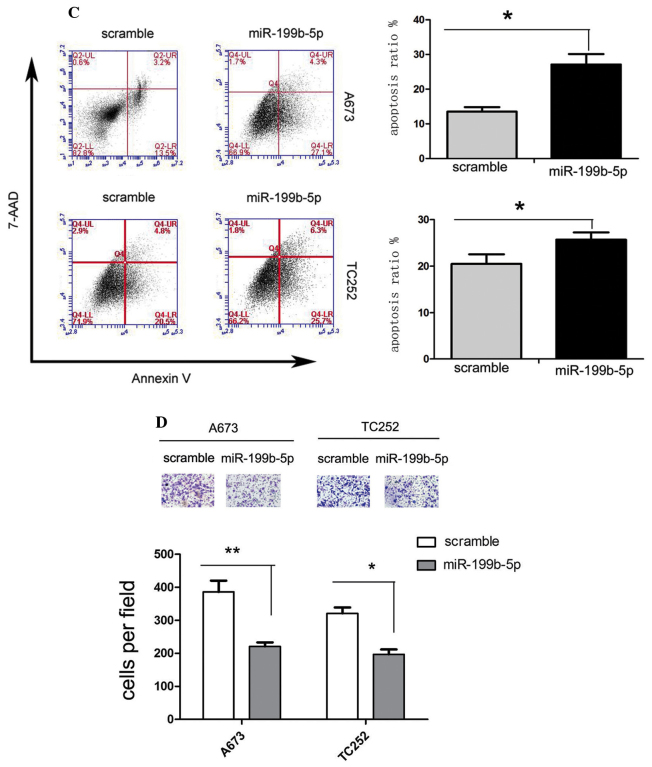
Ectopic expression of miR-199b-5p inhibits cell proliferation, inhibits the cell cycle at the G1- to S-phase transition, induces cell apoptosis and suppresses cell invasion in ES cells. (A) Expression of miR-199b-5p significantly inhibited the cell proliferation in A673/TC252 cells. (B) Flow cytometry was performed to assess the cell cycle and revealed that miR-199b-5p arrested cells at the G1- to S-phase transition. (C) An apoptosis assay demonstrated that miR-199b-5p induced cell apoptosis. In the dot plots, top left quadrant : dead cells; bottom left quadrant: living cells; bottom right quadrant: cells in early apoptosis; and top right quadrant: cells in late apoptosis. (D) An invasion assay revealed that miR-199b-5p suppressed the cell invasion ability. miR, microRNA.

The A673 and TC252 cells were harvested separately following transfection with miR-199b-5p mimic and scramble control at 0, 24, 48 and 72 h. The activity of A673 cells was subsequently assessed at different time points. The overexpression of miR-199b-5p significantly inhibited the cell proliferation compared with the scramble control *in vitro*, as shown in Fig. 2A.

Since cell proliferation was directly associated with the cell cycle, the effect of the miR-199b-5p mimic on the cell cycle was analysed. The quantity of cells in the G1 phase increased significantly following the forced expression of miR-199b-5p (Fig. 2B). By contrast, the percentage of cells in S phase decreased in the A673 and TC252 cells (Fig. 2B). Therefore, the G1- to S-phase transition was inhibited by the overexpression of miR-199b-5p.

Cell apoptosis may be the cause of the change in cell growth and proliferation in the ES cells. The number of early apoptosis cells following transfection with the miR-199b-5p mimic was then assessed. The ratio of early apoptotic cells markedly increased, as detected by PE-Annexin V staining following transfection with the miR-199b-5p mimic compared with the scramble (Fig. 2C).

In addition, the cell invasive ability was determined following the forced expression of miR-199b-5p in ES cells. The Matrigel invasion chamber assays demonstrated that the invasive ability of cells with overexpression of miR-199b-5p significantly decreased (Fig. 2D). These results suggested that miR-199b-5p markedly inhibited cell proliferation, inhibited the cell cycle transition, induced cell apoptosis and suppressed the invasion of ES cells.

### miR-199b-5p represses CCNL1 to regulate ES cells

**Figure 3:**
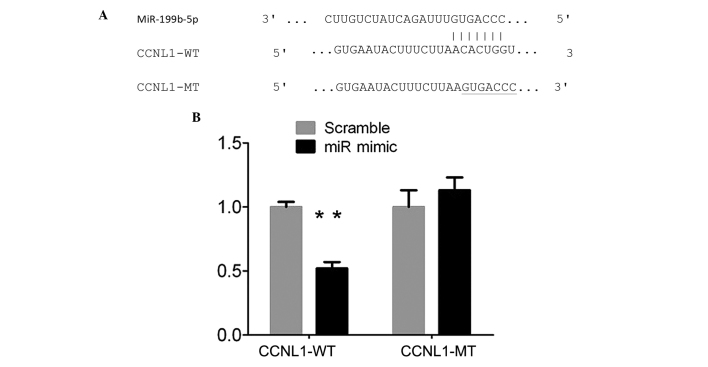
CCNL1 is the target gene of miR-199b-5p *in vitro*. (A) The nucleotide sequences of miR-199b-5p and the complementary sequence in CCNL1 mRNAs revealed the potential binding site. (B) The luciferase activity was performed in the transfected 293T cells and demonstrated a significant decrease in the mRNA expression levels of CCNL1. The miR-199b-5p mimic significantly suppressed the luciferase activity of the reporter gene, containing CCNL1-WT, however, not CCNL1-MUT. The data are expressed as the mean ± standard deviation of three independent experiments (^*^P<0.05, ^**^P<0.01, compared with the scramble control). miR, microRNA; WT, wild-type; MT, mutant.

miRNAs regulate mRNA targets by inhibiting protein translation or directly degrading the mRNA. Therefore, the present study determined potential target genes of miR-199b-5p using Targetscan (www.targetscan.org) and PicTar (www.picTar.mdc-berlin.de). CCNL1 was revealed as a possible target gene of miR-199b-5p in ES cells using a luciferase activity assay. Sequence analysis demonstrated that the 3′UTR of CCNL1 contained the miR-199b-5p binding sites (Fig. 3A). The 3′UTR of CCNL1 was cloned downstream of the pMIR- report gene, which formed CCNL1-WT and similarly, the CCNL1-MT was generated. The results revealed that the relative luciferase activity of the CCNL1-WT was significantly inhibited following transfection with the miR-199b-5p mimic compared with scramble control. However, luciferase activity of the CCNL1-MT remained unchanged (Fig. 3B). Additionally, miR-199b-5p may act as a tumor suppressor by repressing the expression of CCNL1 in ES cells.

### CCNL1 is the target gene of miR-199b-5p

**Figure 4:**
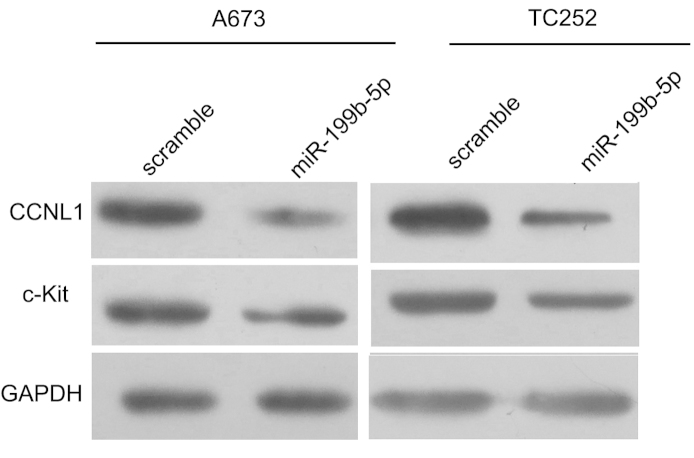
CCNL1 is the target gene of miR-199b, confirmed by western blotting. Western blotting demonstrated the protein expression levels of CCNL1 in the A673/TC252 cell lines following transfection with the miR-199b-5p mimic for 72 h. miR, microRNA.

To confirm that CCNL1 was a target gene of miR-199b-5p, the A673 and TC252 cells were collected and the total protein was extracted following the overexpression of miR-199b-5p at 72 h. Western blotting revealed that the protein expression levels of CCNL1 markedly decreased (Fig. 4A). Therefore, miR-199b-5p may inhibit ES cells by targeting CCNL1 *in vitro*.

## Discussion

miRNAs are important in the progression of tumor cells (13). The expression of miR-199b-5p was downregulated in a wide variety of tumor types, including ovarian cancer, breast cancer, thyroid cancer and osteosarcoma. miR-199b-5p may be downregulated by activation of the JAG1-Notch1 signaling pathways in ovarian cancer (14). miR-199b-5p has been demonstrated to inhibit cancer cell migration and colony formation in breast cancer (15) and reduces the proliferation of thyroid follicular cancer cells (16). Won *et al* (17) revealed that miR-199b-5p is involved in the Notch signaling pathway in osteosarcoma and suggested the inhibitor of miR-199b-5p may be a potential treatment strategy to prevent osteosarcoma metastasis. Garzia *et al* (18) demonstrated that the expression of miR-199b-5p correlated with metastasis in medulloblastoma tumor and indicated that miR-199b-5p may be combined with radiation and chemotherapy as an auxiliary treatment to improve the antitumor effect and life quality of patients. These studies provided to suggest the benefit in identifying the role of miR-199b-5p in ES cells.

The present study assessed the expression levels of miR-199b-5p in ES A673 cells. The expression of mature miR-199b-5p in the A673 cells was similar to the result in TC252 cells. In addition, in A673 and TC252 cells the expression of miR-199b-5p was downregulated compared with the levels in human MSCs, indicating that miR-199b-5p may be involved in ES. Functional experiments indicated that the forced expression of miR-199b-5p suppressed cell proliferation rate, cell invasion, arrested the cell cycle and induced cell apoptosis in each ES cell line. Bioinformatic prediction revealed CCNL1 as a predicted target gene of miR-199b-5p.

Notably, CCNL1 was demonstrated as a direct target gene of miR-199b-5p by measuring luciferase activity and protein expression levels. CCNL1, a cell cycle regulatory protein and a potential oncogene, is localized in the 3q25 region and associated with the survival rate of patients with head and neck squamous cell carcinoma. For instance, CCNL1 was expressed and amplified in human head and neck squamous cell carcinoma, and was suggested as an oncogene (19,20).

In conclusion, the present study has demonstrated that miR-199b-5p acted as a tumor suppressor by targeting CCNL1 in ES cell lines. These findings may provide a novel insight into the molecular mechanism underlying human ES. Furthermore, miR-199b-5p may be a novel diagnostic marker or therapeutic target for the treatment of human ES in the future.
